# Synergistic and Additive Interactions in Essential Oils Obtained from Combined Plant Materials: Enhanced Control of Insect Pests

**DOI:** 10.3390/molecules31060945

**Published:** 2026-03-12

**Authors:** Imtinene Hamdeni, Sonia Boukhris-Bouhachem, Mounir Louhaichi, Abdennacer Boulila, Ismail Amri, Juan José R. Coque, Lamia Hamrouni

**Affiliations:** 1 Laboratory of Management and Valorization of Forest Resources, National Institute of Researches on Rural Engineering, Water and Forests, P.B. 10, Ariana 2080, Tunisia; bouhachems@gmail.com (S.B.-B.); ismail.amri@cnstn.rnrt.tn (I.A.); hamrounilam@yahoo.fr (L.H.); 2 National Institute of Agricultural Research (INRAT), Tunis 1004, Tunisia; 3 International Center for Agricultural Research in the Dry Areas (ICARDA), CGIAR Multifunctional Landscapes Science Program, Tunis 1004, Tunisia; m.louhaichi@cgiar.org; 4 Laboratoire des Substances Naturelles, Institut National de Recherche et d’Analyse Physico-Chimique, Biotechpole of Sidi Thabet, Ariana 2020, Tunisia; abdennacer.boulila@inrap.rnrt.tn; 5 Laboratory of Biotechnology and Nuclear Technology, National Center for Nuclear Sciences and Technologies (CNSTN), Sidi Thabet Technopark, Sidi Thabet 2020, Tunisia; 6 Instituto de Investigación de la Viña y el Vino, Escuela de Ingeniería Agraria y Forestal, Universidad de León, 24009 León, Spain; jjrubc@unileon.es

**Keywords:** essential oils, combined plant material, insecticidal activity, synergism, additivity, multivariate analysis

## Abstract

Essential oils (EOs) from combined plant materials offer a promising alternative to conventional extraction by enhancing chemical diversity and bioactivity. This study evaluated the chemical composition and insecticidal properties of individual and combined plant EOs from *Cymbopogon citratus*, *Eucalyptus camaldulensis*, *Eucalyptus lehmannii*, *Salvia rosmarinus* and *Thymus vulgaris* were evaluated against aphids. Binary and ternary combinations were prepared in equal proportions prior to hydrodistillation. GC-MS analysis revealed significant compositional shifts in EOs from combined plant materials. Major compounds in individual oils included citral (53.11%) and neral (29.14%) in *C. citratus*, thymol (70.84%) in *T. vulgaris*, and eucalyptol as the predominant compound in *E. camaldulensis* (66.51%), *E. lehmannii* (56.99%) and *S. rosmarinus* (46.56%), respectively. In the combined oils, the relative abundance of these constituents was altered, and in some cases new constituents were introduced. Principal Component Analysis (PCA) and Hierarchical Cluster Analysis (HCA) revealed that combined plant EOs clustered near their parental oils, indicating compositional inheritance. Contact toxicity assay against *Aphis fabae* demonstrated enhanced efficacy of the combined oils, with reduced LC_50_ values (1.39 µL mL^−1^ for *E. camaldulensis* + *T. vulgaris*) and synergistic interactions, indicated by a co-toxicity coefficient (CTC) of 221.58 and elevated synergistic factors. Pearson correlation analysis and Partial Least Squares (PLS) regression jointly identified Acorenone B and thymol as negatively, and caryophyllene as positively correlated compounds, all with relatively high contribution to insecticidal activity, ranking highest with a Variable Importance in Projection (VIP) scores > 1.0. While PLS model had modest predictive power, the integration of these statistical approaches supports the insecticidal potential of combined plant-derived EOS in laboratory bioassays and indicates their relevance to sustainable crop protection.

## 1. Introduction

The combination of essential oils (EOs) from different plant species, either through conventional blending of extracted oils, or through the distillation of combined plant materials, introduces a complex chemical matrix that can profoundly influence their biological activities [1,2]. These combinations can lead to specific interactions among the constituent compounds [3]. Additive effects occur when the combined bioactivity matches the cumulative activity of the individual oils [4]. In contrast, antagonistic interactions happen when one or more components reduce the mixture’s efficacy [5]. Synergistic effects are particularly interesting because they produce a combined activity that surpasses the predicted sum of the individual parts [6]. The nature of these interactions depends on several factors, including chemical composition, concentration ratios, and the method used to combine the oils [7]. The distillation of combined plant materials, entailing the simultaneous extraction of multiple botanical sources, can uniquely promote interactions among volatile compounds during the extraction process [8]. This approach has the potential to yield distinct chemical profiles with enhanced antimicrobial activity [9]. Despite its promise, the mechanisms underlying such interactions remain complex and insufficiently elucidated, thereby highlighting the need for further investigation [10].

Interest in EOs as sustainable alternatives to synthetic pesticides has grown due to their broad-spectrum antimicrobial activity and diverse biological targets, which help reduce resistance development in pests and pathogens [11,12,13]. While extensive research has focused on individual EOs and their mixtures, there is comparatively less information on EOs obtained by distilling combined plant materials (CPM-EOs). Unlike post-distillation blends, combining plant materials prior to distillation may alter chemical composition and produce unique synergistic biological effects, highlighting the novelty and necessity of the present study [14,15].

Aromatic and medicinal plants such as *C. citratus*, *S. rosmarinus*, *T. vulgaris*, *E. camaldulensis* and *E. lehmannii* are globally distributed and have been traditionally used for their medicinal and pesticidal properties [16,17]. These species have demonstrated significant antimicrobial and insecticidal potential individually [18]. However, the properties of EOs obtained from their combined plant materials remain underexplored [19]. Leveraging the synergistic potential of these plants species presents promising opportunities for the development of versatile and sustainable biopesticides, tailored to contemporary agricultural systems [20]. Agricultural production across many regions faces serious threats from insect pests including *Aphis fabae* (*A. fabae*), a major pest of legumes that imposes additional pressures on crop health and productivity [21]. *A. fabae* is also an important vector of viruses on potato, pepper and faba bean in Tunisia [22]. Effective biocontrol agents targeting such pests are crucial components of integrated pest management strategies that could include the use of EOS, representing a more sustainable approach to pest control with minimal environmental impact [23,24].

This study investigates EOs extracted from five aromatic species, *C. citratus*, *E. camaldulensis*, *E. lehmannii*, *S. rosmarinus* and *T. vulgaris*. The research focuses on both individual oils and selected binary and ternary combinations, aiming to evaluate how distillation of combined plant materials influences their chemical profiles and biological activities. Chemical composition was analyzed using gas chromatography–mass spectrometry (GC–MS). Insecticidal efficacy was evaluated against *A. fabae* through contact toxicity testing. Principal Component Analysis (PCA) and Hierarchical Cluster Analysis (HCA) were performed on the chemical composition data to evaluate the compositional patterns. In addition, Pearson correlation and Partial Least Squares (PLS) regression were performed to identify the main constituents associated with insecticidal activity based on LC_50_ values. These results contribute to a better understanding of the chemical basis of the bioactivity of combined EOs and support their potential role in sustainable crop protection.

## 2. Results

### 2.1. Chemical Composition of Individual and Combined Plant Material Essential Oils

The chemical analysis of individual and CPM-EOs from *C. citratus*, *E. camaldulensis*, *E. lehmannii*, *S. rosmarinus*, and *T. vulgaris*, as well as their binary and ternary mixtures, led to the identification of 35 compounds, representing 93.66% to 99.99% of the total oil composition. The identified constituents were grouped into four major chemical classes: monoterpene hydrocarbons, oxygenated monoterpenes, sesquiterpene hydrocarbons, and oxygenated sesquiterpenes. All EOs, whether individual or combined, were dominated by oxygenated monoterpenes, ranging from 62.99% to 90.39%, followed by monoterpene hydrocarbons (9.59% to 33.6%) (Table 1).

In *C. citratus* EO, five components accounted for 99.98% of the total oil, primarily oxygenated monoterpenes (90.39%) and monoterpene hydrocarbons (9.59%), with geranial (53.11%), neral (29.14%) and β-myrcene (9.59%) as the most abundant compounds. *E. camaldulensis* EO contained seven constituents (99.92%), dominated by oxygenated monoterpenes (70.79%) and monoterpene hydrocarbons (26.04%), particularly eucalyptol (66.51%) and α-pinene (24.38%). Similarly, *E. lehmannii* EO was rich in oxygenated monoterpenes (74.45%) and monoterpene hydrocarbons (24.78%), with six major chemicals representing 99.6% of the total composition, including eucalyptol (56.99%) and α-pinene (24.18%). *S. rosmarinus* EO comprised ten chemical constituents (99.98%), with eucalyptol (46.56%), borneol (11.35%), and α-pinene (9.06%) as predominant. Finally, *T. vulgaris* EO contained nine components (99.99%), rich in oxygenated monoterpenes (76.56%) and monoterpene hydrocarbons (22.07%), with thymol (70.84%), *p*-cymene (11.01%), and γ-terpinene (7.73%) as the major compounds.

Binary combinations involving *C. citratus* resulted in notable shifts in chemical profiles, reflecting both chemical reorganization and selective transformations depending on the companion species. In the *C. citratus* + *E. camaldulensis* oil, geranial (33.2%) and neral (17.23%) were the main constituents, indicating partial retention of the citrus profile, whereas eucalyptol, characteristic of *E. camaldulensis*, was completely absent. Remarkably, *p*-cymene (19.14%) appeared as a newly formed compound, indicating possible chemical transformation during the distillation process. In the blend with *E. lehmannii*, eucalyptol became the dominant compound (73.62%), a level exceeding that of *E. lehmannii* alone (56.99%) suggesting potential chemical interactions between the matrices during distillation, while geranial and neral decreased sharply to 5.11% and 2.69%, respectively. Additionally, α-pinene declined from 24.18% to 16.9%, reflecting rebalancing of the volatile fraction. The *C. citratus* + *S. rosmarinus* mixture showed a moderate retention of geranial (21.45%) and neral (9.8%), while camphor increased from 7.44% to 9.39%. In contrast, other major *S. rosmarinus* volatiles such as eucalyptol, α-pinene, camphene, and β-pinene were reduced to 26.13%, 3.5%, 2.28%, and 2.9%, respectively. In the *C. citratus* + *T. vulgaris* blend, thymol became the dominant constituent (45.78%), underscoring the strong phenolic imprint of *T. vulgaris*, while geranial (24.12%) and neral (12.34%) were well retained. The appearance of isoneral (2.45%) alongside reduced levels of *p*-cymene (2.92%) and γ-terpinene (3.57%), further highlights the emergence of new volatiles and reorganization of the monoterpene fraction during distillation.

Binary blends containing *E. camaldulensis* exhibited distinct compositional behaviors, ranging from preservation to transformation of key constituents depending on the combined species. Thus, in the *E. camaldulensis* + *E. lehmannii* oil, the major components, eucalyptol (71.27%) and α-pinene (23.39%) were largely retained, indicating compositional stability and chemical compatibility between the two species. Conversely, combination with *S. rosmarinus* led to a profile still dominated by eucalyptol (64.35%) and α-pinene (9.0%), alongside camphor (4.04%), while camphene (2.95%) and β-pinene (2.78%) were preserved at low levels, reflecting moderate rebalancing without major loss of original markers. In contrast, the blend with *T. vulgaris* revealed marked compositional shifts: thymol emerged as the dominant compound (55.69%), slightly reduced from its original concentration in *T. vulgaris* (70.84%). Notably, eucalyptol, originally the primary component of *E. camaldulensis* (66.51%) was no longer detectable, indicating possible chemical transformation. Monoterpene hydrocarbons, including α-pinene and β-pinene were also drastically reduced from 24.38% and 1.21% to 1.88% and undetectable levels, respectively. Meanwhile, *p*-cymene and γ-terpinene present in *T. vulgaris* at 11.01% and 7.73% were enriched to 20.52% and 10.11% respectively. These compositional modifications reflect a clear shift toward the phenolic and aromatic profile of *T. vulgaris* accompanied by a complete attenuation of the eucalyptol-rich signature characteristic of *E. camaldulensis*.

Combinations with *E. lehmannii* resulted in compositional changes influenced by the accompanying species. In the *E. lehmannii* + *S. rosmarinus* blend, eucalyptol remained the predominant compound at 49.62%, reflecting substantial retention from both original oils (56.99% and 46.56% respectively). Camphor, a key constituent of *S. rosmarinus* (7.44%), was slightly reduced to 6.28%, while α-pinene initially abundant in both oils (24.18% and 9.06% respectively) appeared at an intermediate level of 13.77%, indicating a compositional balance between the two sources. In contrast, the combination of *E.* lehmannii with *T. vulgaris* yielded an oil dominated by thymol (35.77%) and eucalyptol (35.09%), both substantially reduced from their original concentrations. *p*-cymene was completely eliminated, α-pinene declined to 11.97%, while γ-terpinene increased slightly from 7.73 to 7.96%, demonstrating a partial merging of the two oils.

Similar compositional patterns were observed in the combination of *S. rosmarinus* with *T. vulgaris*, where thymol (33.56%) and eucalyptol (29.02%) emerged as the predominant compounds shaping the blend’s chemical profile. *p*-cymene was completely eliminated, and α-pinene decreased to 5.09%, reflecting selective changes that enhance the blend’s chemical diversity.

Regarding ternary CPM-EOs, eucalyptol was detected exclusively in *C. citratus* + *T. vulgaris* + *S. rosmarinus*, where it accounted for 10.67%. Thymol, the principal phenolic compound of *T. vulgaris*, consistently dominated all ternary blends, with concentrations varying from 37.64% to 50.46%. *p*-cymene, another major compound from *T. vulgaris*, was present at 16.81% and 3.68% in blends containing *E. camaldulensis* and *E. lehmannii*, respectively, but was entirely absent in *S. rosmarinus* blend. Geranial (15.95–20.9%) and neral (6.43–10.7%) characteristic of *C. citratus* were well preserved across all blends, contributing to their distinctive citrus aroma. Camphor, a marker compound of *S. rosmarinus*, was detected only in its corresponding ternary blend at 3.99%. These compositional variations illustrate the complex interactions occurring in ternary CPM-EOs, where the phenolic dominance of thymol is balanced by the citrus freshness of geranial and neral, while other compounds such as eucalyptol, *p*-cymene, and camphor modulate the aroma profile and potentially the biological activity of the oils.

### 2.2. Multivariate Analysis of Essential Oil Compositions

#### 2.2.1. Principal Component Analysis (PCA)

PCA was performed to explore patterns in the chemical composition of individual and CPM-EOs. The scree plot indicated that the first three principal components account for approximately 95.83% of the total variance, with PC1 explaining 47.13%, PC2 explaining 34.94%, and PC3 contributing an additional 13.76%. Given the sharp decline in eigenvalues after the third component, only PC1 and PC2, together explaining 82.07% of the variance, were considered for graphical representation.

**Figure 1:**
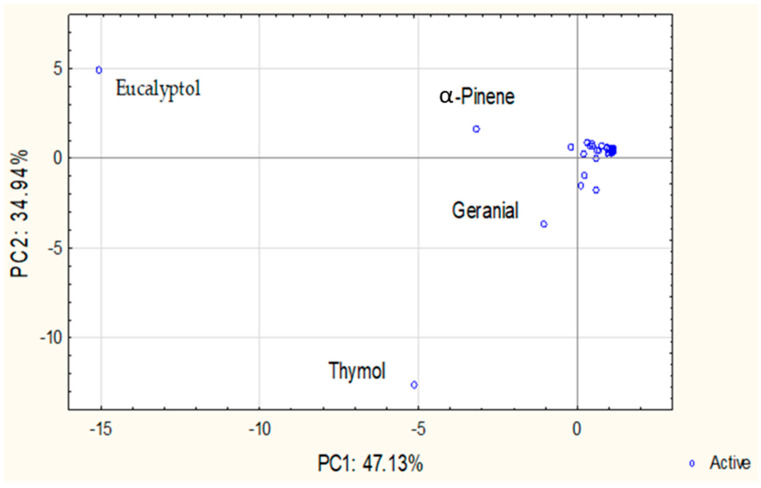
Principal component analysis (PCA) of EOs composition: Projection on PC1 and PC2.

The PCA score plot (Figure 1) revealed distinct groupings driven by the distribution of key chemical constituents in individual and CPM-EOs. Detailed PCA loadings (PC1, PC2 and PC3) for all individual, binary, and ternary CPM-EOs are provided in Supplementary Table S1. *E. camaldulensis* and *E. lehmannii* exhibited strong negative loadings on PC1 (−0.91 and −0.89, respectively), primarily influenced by eucalyptol, a dominant compound strongly correlated with this axis. Similarly, *S. rosmarinus* also loads strongly negatively on PC1 (−0.87) but displays a moderate positive loading on PC2 (0.40), suggesting some chemical similarity with the *Eucalyptus* species while being differentiated by compounds such as camphor, which contributes to its unique profile. *T. vulgaris* showed a strong negative loading on PC2 (−0.88), primarily associated with thymol, underscoring its distinct chemical signature. In contrast, *C. citratus* exhibited moderate loadings near the origin for both PC1 (−0.06) and PC2 (−0.28), indicating a balanced distribution across these components. However, it was primarily characterized by its high loading on PC3 (0.94), driven by its high citral content, which strongly influences this component and distinguishes this species’ chemical profile, reflecting variation that is not fully represented in the PC1/PC2 plot.

The binary and ternary CPM-EOs exhibited clear spatial distributions in the PCA space, shaped by the dominant constituents of their parent oils. Binary combinations involving *Eucalyptus* species, including pairings within the genus and with other plants such as *C. citratus + E. lehmannii* and *E. camaldulensis + S. rosmarinus*, showed strong negative loadings on PC1, reflecting high levels of eucalyptol and α-pinene. Mixtures involving *T. vulgaris*, such as *C. citratus + T. vulgaris* and *E. camaldulensis* + *T. vulgaris* were positioned along PC2 due to thymol content. Combinations with *C. citratus*, especially *C. citratus* + *E. camaldulensis* and *C. citratus* + *S. rosmarinus*, exhibited strong positive loadings on PC3, indicating a distinct geranial-driven profile. Ternary mixtures, such as *C. citratus + T. vulgaris + E. camaldulensis* and *C. citratus + T. vulgaris + E. lehmannii*, loaded strongly on PC2 (thymol-driven), with moderate PC3 contributions reflecting geranial influence. Similarly, *C. citratus + T. vulgaris + S. rosmarinus* showed strong negative loadings on both PC1 and PC2, indicating a combined influence of eucalyptol, α-pinene, and thymol, with minor geranial input. These spatial patterns illustrate the compositional interplay and synergistic effects emerging from plant material combinations.

#### 2.2.2. Hierarchical Cluster Analysis (HCA)

**Figure 2:**
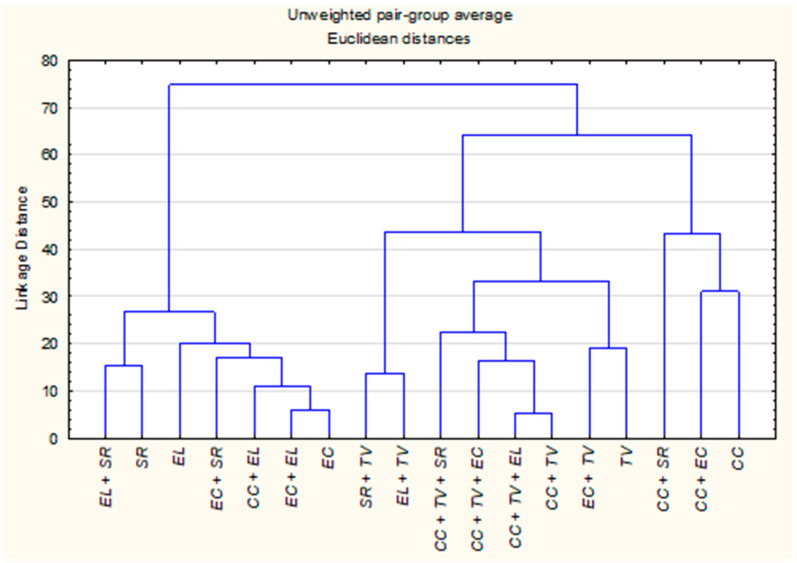
Hierarchical clustering dendrogram of individual and CPM-EOs of *C. citratus* (*CC*), *E. camaldulensis* (*EC*), *E. lehmannii* (*EL*), *S. rosmarinus* (*SR*), and *T. vulgaris* (*TV*) based on their chemical composition. Clustering was performed using Euclidean distances and the UPGMA method.

The HCA dendrogram, constructed using Euclidean distances and the UPGMA method, revealed clear groupings among the EOs and their combinations, in agreement with the patterns observed in the PCA. Three primary clusters emerged based on compositional similarity (Figure 2).

Cluster A consisted of *Eucalyptus* species, *S. rosmarinus* and their mixtures. The close proximity between *E. camaldulensis* and *E. lehmannii* (distance = 16) reflects a high degree of shared constituents, further emphasized by the even closer distance between *E. camaldulensis* and its blend with *E. lehmannii* (distance = 6). Additionally, *E. camaldulensis + S. rosmarinus* (distance = 18 from *E. camaldulensis* and 22.3 from *E. lehmannii*) supports a trend of compositional alignment within this cluster, likely driven by common oxygenated monoterpenes such as eucalyptol and α-pinene. Cluster B was dominated by *T. vulgaris* and its mixtures, characterized by high phenolic content. While *T. vulgaris* alone was chemically distinct from *E. camaldulensis* (distance = 101), their combination showed a greatly reduced distance of 19, indicating a closer chemical relationship. Mixtures involving *C. citratus* and *T. vulgaris* also exhibited strong chemical affinity. For instance, *C. citratus + T. vulgaris* and *C. citratus + T. vulgaris + E. lehmannii* displayed a very low inter-sample distance of 5.4, while *C. citratus + T. vulgaris + E. camaldulensis* showed a slightly higher but still close distance of 17.9. These shifts suggest enhanced chemical integration, likely driven by synergistic interactions between thymol and geranial components. The mixture *E. lehmannii + T. vulgaris* (distance = 46) also supports the potential for moderate compositional blending between phenolic-rich and monoterpene-based oils. Cluster C encompassed *C. citratus* and its combined derivatives. Although *C. citratus* alone was chemically distant from most other oils (distance > 90 from *E. camaldulensis* and *T. vulgaris*), its mixtures consistently reduced inter-sample distances and frequently occupied intermediate positions between Clusters A and B. Notable examples include *C. citratus + E. camaldulensis* (distance = 31 from *C. citratus*), *C. citratus + S. rosmarinus* (48.1) all suggesting partial compositional blending. These results indicate that combining plant material modifies the volatile profile in a manner that reduces chemical distances between initially dissimilar oils.

### 2.3. Insecticidal Activity

#### 2.3.1. Probit Analysis of Individual and Combined Plant Material Essential Oils

Probit regression analysis revealed notable variation in the insecticidal efficacy of both individual and CPM-EOs against *A. fabae* after 24 h of exposure. Among the individual oils tested, *E. camaldulensis* demonstrated the highest insecticidal potency, with the lowest LC_50_ value of 2.45 µL mL^−1^, indicating its efficiency was obtained with the smallest concentration to kill 50% of the aphid population. This was followed by *E. lehmannii* with LC_50_ of 2.90 µL mL^−1^, *C. citratus* at 3.24 µL mL^−1^, and *T. vulgaris* at 3.71 µL mL^−1^. The least effective individual oil was *S. rosmarinus*, which exhibited the highest LC_50_ value of 4.41 µL mL^−1^, reflecting lower toxicity (Table 2).

When evaluating CPM-EOs, binary mixtures generally lead to enhanced insecticidal activity compared to individual ones. The binary combination of *E. camaldulensis* + *T. vulgaris* was the most potent, achieving an LC_50_ of 1.39 µL mL^−1^, indicating good synergy between components inducing a strong increase in toxicity. Other binary mixtures involving *C. citratus* also showed substantial improvements, with LC_50_ values ranging from 1.75 to 2.38 µL mL^−1^, demonstrating a relative synergistic or additive effect. Ternary combinations resulted in complete mortality (100%) at the lowest tested concentration of 2 µL mL^−1^. While this precluded a fitting dose–response curve and calculating LC_50_ values, these results indicate potential synergistic effects among the three oils. However, further testing at lower concentrations would be required to accurately quantify the degree of synergy.

#### 2.3.2. Assessment of Synergistic Interactions Using Co-Toxicity Coefficient and Synergistic Factors

The evaluation of CPM-EOs against *A. fabae* revealed a spectrum of interaction types, ranging from strong synergism to additive or slight antagonism. *C. citratus* stood out as a key synergistic component. Its binary combinations with *E. camaldulensis*, *E. lehmannii*, *S. rosmarinus*, and *T. vulgaris* consistently exhibited strong synergistic effects, with co-toxicity coefficients (CTC) ranging from 130.64 to 198.57 and synergistic factors (SF) exceeding 1.2 in all cases (Table 3).

*T. vulgaris* also contributed significantly to enhanced efficacy when paired with other species. Combinations with *E. camaldulensis*, *E. lehmannii*, and *S. rosmarinus* demonstrated particularly strong synergy, reflected by CTC values greater than 218 and SF values above 2, indicating substantial improvement in insecticidal activity. *S. rosmarinus*, despite being the least toxic as a single oil, displayed notable synergistic effects in combinations. When paired with *C. citratus* or *T. vulgaris*, the mixtures achieved high CTC values (up to 249.08) and strong SF values (>2.2), underscoring its supportive role in enhancing toxicity. However, in combination with *E. camaldulensis*, the interaction was weaker, classified as additive to moderate synergism (CTC = 119.52), and showed uneven SF values (0.85 and 1.54), suggesting variable contribution depending on the pairing. In contrast, the mixture of *E. camaldulensis* and *E. lehmannii* showed the weakest interaction, classified as additive to slight antagonism. This combination yielded a CTC of 81.58 and SF values of 0.75 and 0.88, indicating little to no enhancement and potential interference in insecticidal action.

Ternary mixtures comprising *C. citratus*, *T. vulgaris*, and either *S. rosmarinus* or, an *Eucalyptus* species caused complete mortality at the lowest tested dose (2 µL mL^−1^). This prevented LC_50_ estimation but clearly indicated highly effective interactions. In summary, *C. citratus* and *T. vulgaris* were the primary drivers of synergy across combinations, while *S. rosmarinus* enhanced their effects significantly. Conversely, pairings involving only *Eucalyptus* species provided limited or even slightly antagonistic results, emphasizing the importance of strategic plant material selection for optimal insecticidal efficacy.

#### 2.3.3. Correlation and Regression Analysis Based on LC_50_

To identify the chemical constituents most strongly associated with insecticidal activity, correlation and regression analyses were performed using LC_50_ values as indicators of toxicity. Pearson correlation analysis provided insight into the strength and direction of linear relationships between key constituents and bioactivity. To further explore these associations in a multivariate context and account for potential interactions between compounds, PLS regression was applied. This approach allowed the development of a predictive model and highlighted the key constituents contributing to the overall insecticidal effect of EOs.

##### Pearson Correlation Between Major Constituents and LC_50_ Values

Among the 35 EOs constituents analyzed, three exhibited statistically significant correlations with LC_50_ values. Thymol (r = −0.5018, *p* = 0.034) and Acorenone B (r = −0.5119, *p* = 0.030) were negatively correlated, indicating that higher concentrations of these compounds corresponded to lower LC_50_ values and therefore greater insecticidal activity. Caryophyllene was positively correlated with LC_50_ (r = 0.5267, *p* = 0.025), suggesting that higher concentrations of this compound were associated with higher LC_50_ values and, therefore, reduced insecticidal potency. No other constituent of the EOs showed a statistically significant correlation with LC_50_ values. The complete list of correlation coefficients and *p* values for all analyzed compounds is provided in Table 4.

##### Partial Least Square Regression Modeling

**Figure 3:**
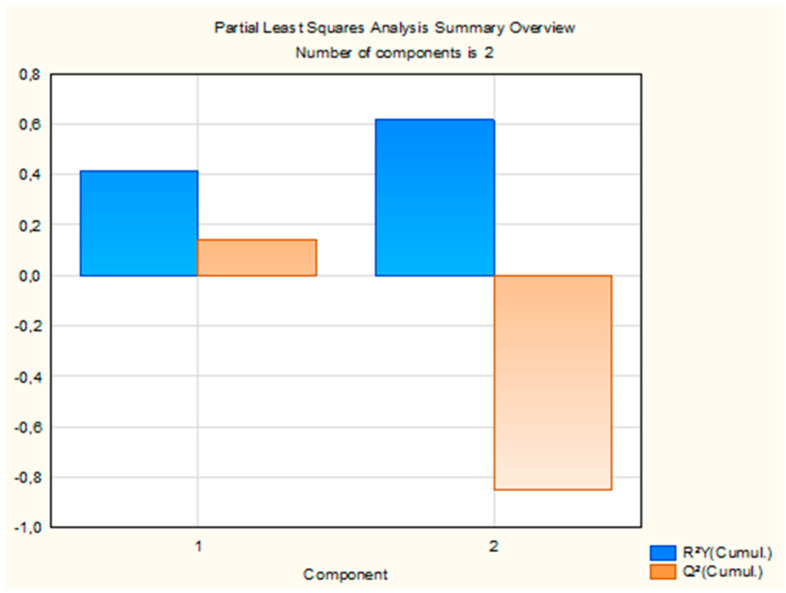
Cumulative R^2^Y and Q^2^ statistics of the two PLS components explaining variance in LC_50_.

The PLS regression model extracted two components, which together explained 61.57% of the variance in insecticidal activity (LC_50_). Detailed statistics for each component, including R^2^X, R^2^Y, eigenvalues, Q^2^, and the number of iterations, are presented in Supplementary Table S2. The first component explained 21.86% of the variance in the predictor variables (R^2^X) and 41.62% of the variance in the response variable (R^2^Y), with an eigenvalue of 6.73. The associated Q^2^ value was 0.14, indicating moderate predictive accuracy. This component was statistically significant. The second component contributed 10.61% to R^2^X, bringing the cumulative R^2^X to 32.47% and R^2^Y to 61.57%. Its eigenvalue was 1.91, but the Q^2^ value was negative (−1.15) and the component was not statistically significant. Although the cumulative R^2^Y increased with the addition of the second component, the corresponding negative Q^2^ value indicates limited predictive contribution beyond the first component, which therefore dominates the predictive performance of the model. The overall performance of the model is summarized in Figure 3.

**Figure 4:**
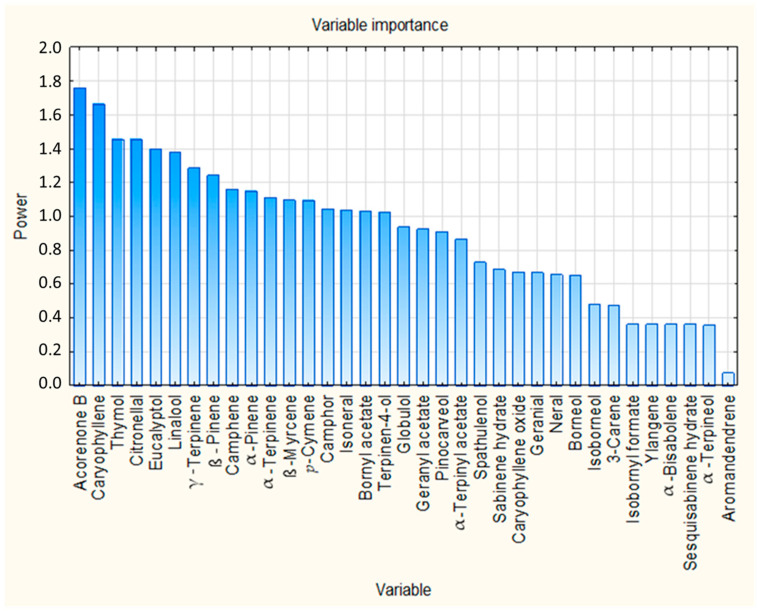
VIP scores of all identified chemical compounds from PLS regression analysis.

The VIP scores from the PLS regression model were used to assess the relative importance of the 35 identified compounds in predicting insecticidal activity. VIP scores quantify the contribution of each chemical constituent used as a predictor variable to explain variation in the response variable, and compounds with VIP scores greater than 1 are considered contributors to the model. As shown in Figure 4, 17 compounds exceeded the commonly used threshold of VIP > 1, indicating relatively high contribution to the model. These 17 compounds are summarized in Table 5, ranked according to their VIP scores. The complete list of compounds and their associated VIP scores are presented in Supplementary Table S3.

Acorenone B (VIP = 1.762), caryophyllene (VIP = 1.663) and thymol (VIP = 1.457) were identified as the compounds with the highest VIP scores, indicating their relatively high influence on the PLS regression model. It is noteworthy that these same compounds also showed statistically significant correlations with insecticidal activity in the Pearson correlation analysis presented previously. The convergence of the results of these two independent statistical approaches reinforces their biological relevance and underlines their role as contributors to the observed insecticidal effect. Besides these compounds, several other compounds with VIP greater than 1, such as citronellal, eucalyptol, linalool, and γ-terpinene, also showed notable importance in the model and could contribute through additive or synergistic interactions. This observation supports the notion that insecticidal activity arises from the collective contribution of multiple constituents, rather than being solely driven by a single dominant compound.

## 3. Discussion

The chemical composition of EOs is a primary determinant of their biological activity, and in this study, the profiles of the tested EOs largely conformed to established chemotypes. Citral is the primary component of EOs derived from *Cymbopogon* species, with the present study showing high levels of geranial (53.11%) and neral (29.14%), closely aligning with Brasilian (geranial 55.48%, neral 35.40%) and Egyptian (geranial 31.57%, neral 13.42%) chemotypes [25,26]. Similar citral-dominant profiles have also been reported in Indonesian [27], Indian [28], and Vietnamese [29] *C. citratus* oils, confirming the consistency of this chemotype across regions. *E. camaldulensis* and *E. lehmannii* were both dominated by eucalyptol, consistent with previous findings, where eucalyptol typically ranges from 20 to 40% [30,31]. However, eucalyptol was more abundant in our samples (50–70%). Variability in chemical composition of *Eucalpytus* EOs can arise from geographic origin, climate, soil conditions, harvesting time, and extraction method [32,33]. *S. rosmarinus* EO was mainly composed of eucalyptol (46.56%) indicating a predominance of eucalyptol chemotype [34,35,36], though other chemotypes such as camphor [37], verbenone [38], α-pinene [39] and myrcene [40] have been described. *T. vulgaris* EO was characterized by a predominance of thymol, consistent with the literature [41,42], but with a higher proportion of thymol (reaching 70%) than previously reported (close to 40%) [43,44,45]. Additional chemotypes for *T. vulgaris* EO were reported including carvacrol [46], linalool [47] and α-terpineol [48].

Upon combining and distilling plant materials, the resulting EOs showed strong compositional inheritance from their respective parent profiles, as indicated by the clustering of major components. However, notable differences were observed, including suppression of certain constituents, emergence of new compounds, and significant shifts in relative abundance. In particular, combinations involving *C. citratus* with *Eucalyptus* species showed distinct profiles, likely due to the interaction of geranial and eucalyptol, whereas blends with *T. vulgaris* were dominated by thymol, reflecting its strong phenolic character. Chemically similar oils, such as *E. camaldulensis*, *E. lehmannii* and *S. rosmarinus*, showed limited differentiation, suggesting lower synergistic effects. These observations highlight the importance of strategic EO selection in combined distillation to achieve both compositional and functional diversity. Similar transformations were previously observed during the combined plant material distillation of *C. citratus* and *Hyptis suaveolens*, where new major constituents (piperitone (40.8%) and p-menth-4(8)-ene (13.2%)) appeared, while β-pinene, sabinene, neral and geranial disappeared, and limonene and α-pinene increased. These mechanisms are inferred from analytical evidence and literature precedent and were attributed to thermal and acid-catalyzed reactions, including cyclization, isomerization, and dehydration, leading to the conversion of citral isomers (neral and geranial) to piperitone, rearrangement of sabinene to p-menth-4(8)-ene and the transformation of myrcene and β-pinene into limonene [2]. Additionally, Sánchez-Velandia et al. [49] reported that in situ chemical reactions can occur during distillation, for example, α-pinene undergoes acid-catalyzed rearrangement to camphene, which is further oxidized to camphor, while myrcene, formed via β-pinene pyrolysis, can be converted to d-citronellal and ultimately cyclized to produce l-menthol. Consistent with these observations, in the present study, these compounds were not detected in the individual plant distillates processed under the same conditions, suggesting that contamination is unlikely. These transformations demonstrate how naturally occurring compounds can undergo significant structural changes during extraction and processing [50]. These reactions can generate new compounds not originally present in the biomass, influencing both chemical complexity and biological activity, with implications for therapeutic, antimicrobial, or preservative effects [51,52]. While these findings strongly suggest these reaction pathways, targeted experimental studies would be required to definitively confirm them.

These compositional changes arise from multiple chemical and physical factors inherent to the distillation process [53,54]. Hydrodistillation exposes EO constituents to elevated temperatures, prolonged contact with water, and oxygen, promoting chemical transformations [55,56]. Thermal degradation particularly affects heat-sensitive compounds like monoterpenes and sesquiterpenes, which can volatilize, decompose, or rearrange [57,58]. Hydrolysis can cleave esters and glycosides, generating alcohols and acids, with different volatilities and polarities, affecting extraction efficiency and oil composition [59,60]. Oxidative reactions, driven by dissolved oxygen or trace metals, can transform reactive aldehydes, phenolics, or unsaturated compounds into oxidized derivatives, that are not present in the raw plant material [61,62,63]. Physical factors such as differences in vapor pressure cause more volatile compounds to distill preferentially, potentially suppressing less volatile components or causing uneven release [64]. Moreover, the extraction procedure can influence physical properties such as viscosity, coloring, and odor, which may affect EOs quality [65]. When different plant matrices are combined, their anatomical and microstructural differences, such as trichome density, oil gland structure, and moisture content, can influence the release kinetics of EO constituents, leading to suppression or enhancement of certain compounds [66].

The insecticidal assay against *A. fabae* revealed variable toxicity levels among the tested oils. Among the individual ones, *E. camaldulensis* exhibited the highest toxicity, followed by *E. lehmannii* consistent with literature attributing strong insecticidal activity to eucalyptol-rich oils [67]. For instance, Khedhri et al. [68] demonstrated potent toxicity of four *Eucalyptus* species against *A. fabae* with LC_50_ ranging from 0.264 to 0.39 mg mL^−1^. Differences in LC_50_ units (mg mL^−1^ in Khedhri et al. vs. µL mL^−1^ in the present study) and variations in EO density should be taken into account when interpreting this comparison. *C. citratus* EO also showed considerable activity, likely due to its high content in geranial and neral, which have been reported to be strong insecticidal and repellent compounds against *A. fabae* [69]. Comparable efficacy was observed against related species including *A. gossypii*, *A. citricola* and *M. persicae*, further supporting the broad-spectrum activity of these oxygenated monoterpenes against the Aphididae family [69,70,71,72]. Similarly, *T. vulgaris* EO exhibited notable insecticidal activity which is attributed to its high thymol content, a monoterpene phenol with well-documented neurotoxic effects on insects [73]. In aphid species, several mechanisms have been described for thymol, including modulation of GABA-gated chloride channels and interference with the tyramine–octopamine signaling pathway, both of which are crucial for neural transmission and behavioral regulation in insects [74]. In the present study, *S. rosmarinus* EO exhibited the lowest activity, with LC_50_ value of 4.41 µL mL^−1^. These results are consistent with those obtained by Casas et al. [75] who reported a similar LC_50_ value (4.61 µL mL^−1^) against *Myzus persicae*.

When EOs were combined, they generally exhibited enhanced toxicity when compared to individual oils. For instance, *E. camaldulensis* + *T. vulgaris* mixture demonstrated the lowest LC_50_ value of 1.39 µL mL^−1^. The CTC and SF analyses confirmed strong synergy in combinations involving *C. citratus*, *T. vulgaris*, and *S. rosmarinus*. Statistical analyses identified acorenone B, thymol and caryophyllene as compounds with relatively high contributions to the insecticidal activity. In agreement, acorenone B has been reported to inhibit both acetylcholinesterase and butyrylcholinesterase, with IC_50_ values of 40.8 µg mL^−1^ and 10.9 µg mL^−1^ respectively, indicating a neuroinhibitory mode of action that disrupts cholinergic neurotransmission and leads to paralysis and death in insects. Consistently, acorenone-rich EOs exhibited high ecotoxicity, supporting the contribution of acorenone-type sesquiterpenes to EO-mediated toxicity [76]. These results are in line with the findings of Bora et al. [77], who reported that multi-component EO blends with different mechanism of action enhance pest control efficiency and delay resistance development. In line with these observations, a study on *Sitophilus zeamais* demonstrated that optimized mixtures of plant volatile compounds, including thymol, carvacrol, phellandrene, γ-terpinene, pulegone, and δ-3-carene, exhibit synergistic insecticidal activity through multitarget effects, impacting neurotransmission and reducing detoxification enzyme activities [78]. Similar synergism among the major constituents (Eucalyptol, carvacrol, pulegone, and eugenol) of *Rosmarinus officinalis*, *Origanum compactum*, *Mentha pulegium*, *Thymus satureioides*, *Myrtus communis* and *Eugenia aromatica* against *Callosobruchus maculatus*, has been shown to improve stability, absorption, cuticular penetration and multi-target neurophysiological effects [79]. Binary mixtures of thymol and eucalyptol demonstrated synergistic toxicity against *Helicoverpa armigera*, inhibiting detoxification and neurophysiological enzymes more effectively than single compounds [80]. Modeling-based research on *Musca domestica* demonstrated that the insecticidal activity of thyme EOs is primarily attributed to thymol, *p*-cymene and γ-terpinene, identified as the main active constituents through component effect analysis. Mixture modeling confirmed synergistic interactions among all binary combinations of these compounds, with the optimal ternary ratio (*p*-cymene: γ-terpinene: thymol = 32:23:45) achieving the highest mortality rate of 87.5%. This optimized blend significantly outperformed individual components, underscoring the crucial role of compositional optimization and synergistic interactions among terpenoid constituents in enhancing the efficacy and consistency of EO-based insecticides [81]. Several reports have highlighted the insecticidal potential of caryophyllene [82,83,84,85]. For example, Sun et al. [86] demonstrated that caryophyllene, α-pinene and β-myrcene act as major bioactive components responsible for the insecticidal and repellent activity of *Peucedanum terebinthinaceum* EO. Additionally, Chohan et al. [87] reported that β-caryophyllene and α-pinene exhibit strong fumigant toxicity against *Myzus persicae*, significantly affecting genes responsible for reproduction, dispersion, and insect growth. Moreover, β-caryophyllene-rich leaf EO of *Psidium guajava* showed strong contact toxicity against *Sitophilus zeamais*, highlighting the eco-friendly potential of this compound as natural insecticide and control agent [88].

## 4. Materials and Methods

### 4.1. Plant Material

During the spring of 2025 (March–April), five plant species (*C. citratus*, *E. camaldulensis*, *E. lehmannii*, *S. rosmarinus* and *T. vulgaris*) were collected from various regions across Tunisia. Leaves were obtained from *C. citratus*, *E. camaldulensis* and *E. lehmannii*, while the aerial parts were gathered for *S. rosmarinus* and *T. vulgaris*. *C. citratus* was cultivated, and the other species were collected from the wild. To ensure representative sampling, plant materials were randomly harvested from several individual plants or trees within each species. The samples were then combined to form homogenized samples, as summarized in Table 6. After collection, the plant materials were placed in a glass greenhouse and air-dried under ambient conditions for five days. Once dried, they were stored in paper bags at room temperature until further analysis. All plant species were taxonomically identified by Professor Lamia Hamrouni following standard procedures. Corresponding voucher specimens have been deposited in the herbarium division of the National Institute of Research on Rural Engineering, Water and Forests (INRGREF).

### 4.2. Essential Oil Extraction and Combinations

EOs were extracted by hydro-distillation of 50 g of dried plant material using a clevenger-type apparatus (SAF Wärmetechnik LabHEAT^®^ KM-ME, 1000 mL, SAF GmbH, Hamm, Germany) with 500 mL distilled water at a plant material-to-water ratio of 10:1 (*v*/*w*) for 3 h [89]. Extractions were performed on individual species (*C. citratus*, *E. camaldulensis*, *E. lehmannii*, *S. rosmarinus*, and *T. vulgaris*) as well as on binary (1:1) and ternary (1:1:1) mixtures, prepared by mixing the dried materials in equal weight ratios prior to distillation [9]. Extractions were performed in three replicates. The extracted oils were dried over anhydrous sodium sulfate and stored at 4 °C in amber glass vials until further use. The EO yield was calculated as % (*w*/*w*) relative to the dry weight of plant material and is reported in Table 1. The overall experimental design is shown in Supplementary Figure S1.

### 4.3. Gas Chromatography Mass Spectrometric (GC-MS) Analysis

The GC-MS analysis was conducted using an HP 8890 gas chromatograph coupled to an HP 5977B mass spectrometer equipped with an HP-5MS UI column (30 m × 0.25 mm; 0.25 µm) (Agilent Technologies, Santa Clara, CA, USA). The injector and the detector temperatures were set at 250 °C and 280 °C, respectively. The oven temperature was programmed from 45 °C (held 1 min) to 250 °C (held 6 min) at a rate of 5 °C/min, for a total runtime of 48 min. Helium was used as the carrier gas at a constant flow rate of 1 mL min^−1^. Samples (1 µL) were injected in split mode with a 1:100 ratio. Mass spectrometry data were acquired in scan mode over a mass range of *m*/*z* 30 to 600. Components were identified by comparing their calculated retention indices (RI calc.), determined relative to a homologous n-alkane series, with literature retention indices (RI lit.), and by comparison of their mass spectra with those present in commercial libraries (Wiley 7, NIST 05) and/or reported in the literature [51].

### 4.4. Evaluation of Insecticidal Activity

#### 4.4.1. Aphid Sampling and Rearing

Pathogen-free apterous parthenogenetic populations of *A. fabae* were collected from faba bean (*Vicia faba*) crops in the Cap Bon region (36.69° N, 10.49° E). Colonies were maintained on potted faba bean plants in a controlled-environment growth chamber at 23 °C ± 1, a relative humidity 60 ± 5%, and a 16:8 h light:dark photoperiod [67]. Aphids and host plants were kept in insect-proof cages equipped with fine mesh vents to ensure proper ventilation and prevent contamination.

#### 4.4.2. Contact Toxicity Bioassay

The insecticidal activity of individual and CPM-EOs was evaluated against *A. fabae* using a direct contact toxicity test. The assays were conducted using concentrations of 2, 4, 6, 8, 10, and 12 μL mL^−1^ [90,91]. For each treatment, an emulsion was prepared by mixing the appropriate volume of EO with 1% (*v*/*v*) Tween 20 solution (Sigma-Aldrich, St. Louis, USA, P1379). Ten wingless aphids of similar size and developmental stage were gently transferred onto a fresh faba bean leaf placed on Whatman filter paper disks inside a sterile 90 mm Petri dish. All individuals were adult females, consistent with the parthenogenetic biology of *A. fabae*. Each group was then sprayed with 1 mL of the prepared EO emulsion, ensuring even coverage of both aphids and the leaf surface. Control groups were treated with 1 mL of 1% Tween 20 solution without EOs. No mortality was observed in the control, so Abbott’s correction was not required before estimating LC_50_ values. All treatments, including the control, were replicated five times. Mortality was assessed 24 h post-application. Aphids were considered dead if they showed no movement of legs or antennae upon gentle probing with a fine brush. Lethal concentration value (LC_50_) with 95% confidence interval was estimated using PROBIT analysis.

#### 4.4.3. Interaction Assessment

The co-toxicity coefficient (CTC) was calculated to evaluate the interaction effects of CPM-EOs on *A. fabae* toxicity. For each mixture, the expected LC_50_ was calculated as the arithmetic mean of the LC_50_ values of the individual EOs. The CTC was then determined by dividing the expected LC_50_ by the observed LC_50_ of the mixture and multiplying by 100. A CTC value greater than 120 was considered indicative of strong synergism, values between 80 and 120 indicated additive (cumulative) effects, and values less than 80 suggested antagonism [92]. Additionally, the synergistic factor (SF) was calculated to assess further the interaction between EOs mixtures [93]. The SF was determined separately for each component of the mixture by dividing the LC_50_ of the individual EO by the LC_50_ of the corresponding mixture. Specifically, SF vs. A = LC_50_ of EO A/LC_50_ of the mixture, and SF vs. B = LC_50_ of EO B/LC_50_ of the mixture. An SF value greater than 1 indicated synergism, a value equal to 1 indicated an additive effect, and a value less than 1 suggested antagonism.

For interaction classification, CTC was used as the primary indicator of overall interaction, while SF values were used to refine the interpretation. When SF values were mixed (one SF > 1 and one < 1) and the CTC fell within the additive range (80–120), interactions were classified based on the position of the CTC within this range: values closer to 120, together with at least one SF > 1, were interpreted as additive to moderate synergism, whereas values closer to 80, together with SF values < 1, were interpreted as additive/slight antagonism. Accordingly, CTC and SF were interpreted jointly to ensure a consistent and transparent classification of interaction effects, particularly in borderline cases.

### 4.5. Statistical and Chemometric Analysis

All analyses were performed using STATISTICA software version 10 (StatSoft, Tulsa, OK, USA). EO yields were determined in three replicates and expressed as mean ± standard deviation. One-way ANOVA followed by Tukey’s HSD test (*p* < 0.05) was used to compare yields. Insecticidal bioassays were conducted in five replicates, and Probit analysis was performed to estimate lethal concentration (LC_50_) with 95% confidence interval. Principal Component Analysis (PCA) and Hierarchical Cluster Analysis (HCA) were conducted to examine patterns in the chemical composition of individual and CPM-EOs. HCA was based on Euclidean distance and clustering was performed using the Unweighted Pair Group Method with Arithmetic Mean (UPGMA). To study the relationship between EO composition and insecticidal activity, chemometric approaches were used. Pearson correlation analysis identified significant linear associations between the abundance of individual compounds and their insecticidal effects based on LC_50_ values. In addition, PLS regression was used to model the relationship between the chemical profiles (X variables) and insecticidal activity (Y variable). Prior to analysis, X variables were scaled to unit standard deviation, and the optimal number of components was determined by cross-validation to minimize prediction error and avoid overfitting. The VIP (Variable Importance in Projection) scores of the PLS model highlighted compounds with relatively high contributions to the activity, acknowledging that the model’s predictive power was modest. These multivariate chemometric analyses provided a better understanding of the associations between the chemical profiles of EOs and their insecticidal efficacy.

## 5. Conclusions

The combination of plant material facilitates effective compositional blending, as evidenced by multivariate analyses that revealed distinct chemical profiles predominantly influenced by key constituents such as eucalyptol, thymol, and geranial. These compositional alterations were observed in the insecticidal bioassay against *A. fabae.* Among the individual EOs tested, *E. camaldulensis* exhibited the highest toxicity. Notably, combinations involving *C. citratus*, *Eucalyptus* species and *T. vulgaris* demonstrated pronounced synergistic effects, significantly increasing toxicity and reducing LC_50_ values, with the *E. camaldulensis* + *T. vulgaris* mixture being the most effective. Althought, *S. rosmarinus* displayed the lowest individual toxicity, its inclusion in blends contributed positively to overall insecticidal efficacy, whereas, combinations composed solely of *Eucalyptus* species exhibited limited or mildly antagonistic interactions. Multivariate analysis further identified acorenone B, thymol and caryophyllene as compounds with relatively high contributions to the insecticidal activity. The observed activity may involve multiple mechanisms, including neurophysiological disruption and interference with reproductive, developmental, and behavioral processes, potentially enhanced by synergistic interactions. Although based on laboratory assays with a single pest species and no assessment of non-target organisms, these results support the potential of these bioactive-enriched EOs as environmentally sustainable alternatives to conventional synthetic insecticides. Further research is warranted to optimize formulation strategies, evaluate field-level applications, investigate the molecular mechanisms that may underlie their bioactivity, and test lower concentrations to accurately quantify their toxicity and potential insecticidal effects.

## Figures and Tables

**Table 1:** Chemical composition (%) and yield (% *w*/*w*, dry basis) of individual and CPM-EOs of *C. citratus* (*CC*), *E. camaldulensis* (*EC*), *E. lehmannii* (*EL*), *S. rosmarinus* (*SR*), and *T. vulgaris* (*TV*) identified by GC-MS analysis.

	Yield (%)								CPM-EOs												
				Individual					Binary										Ternary		
No	Compounds	RI calc.	RI lit.	*CC*	*EC*	*EL*	*SR*	*TV*	*CC* + *EC*	*CC* + *EL*	*CC* + *SR*	*CC* + *TV*	*EC* *+* *EL*	*EC* *+* *SR*	*EC* *+* *TV*	*EL* *+* *SR*	*EL* *+* *TV*	*SR* *+* *TV*	*CC* *+* *TV* + *EC*	*CC* *+* *TV* + *EL*	*CC* *+* *TV* + *SR*
1	α-Pinene	916	928	-	24.38	24.18	9.06	1.11	0.8	16.9	3.5	0.66	23.39	9	1.88	13.77	11.97	5.09	0.77	0.71	1.57
2	Camphene	931	948	-	-	-	4.85	-	-	-	2.28	-	-	2.95	-	4.54	-	3.09	-	-	0.82
3	β-Pinene	960	974	-	1.21	-	6.64	-	-	-	2.9	-	-	2.78	-	2.98	0.39	3.37	-	-	0.98
4	β-Myrcene	977	988	9.59	-	-	2.39	1.25	5.91	-	2.93	2.34	-	0.9	1.09	1.51	0.75	2.23	1.03	2.19	1.7
5	3-Carene	994	1008	-	0.45	-	-	-	-	-	-	-	-	-	-	-	-	-	-	-	-
6	α-Terpinene	1007	1017	-	-	-	-	0.97	-	-	-	0.53	-	-	-	-	-	0.79	-	0.54	-
7	*p*-Cymene	1019	1020	-	-	-	-	11.01	19.14	-	-	2.92	-	-	20.52	-	-	-	16.81	3.68	-
8	Eucalyptol	1020	1031	-	66.51	56.99	46.56	-	-	73.62	26.13	-	71.27	64.35	-	49.62	35.09	29.02	-	-	10.67
9	γ-Terpinene	1052	1059	-	-	-	-	7.73	-	-	-	3.57	-	-	10.11	-	7.96	6.01	3.38	3.85	4.94
10	Sabinene hydrate	1078	1065	-	-	12.39	1.25	-	-	-	0.52	-	-	-	-	9.42	0.78	1.18	-	-	-
11	Linalool	1112	1099	-	-	-	-	2.71	0.92	-	1.31	2.01	-	-	1.27	-	-	-	0.57	1.84	1.22
12	Pinocarveol	1144	1140	-	0.72	-	-	-	-	-	-	-	-	-	-	-	1.06	-	-	-	-
13	Camphor	1145	1143	-	-	-	7.44	-	-	-	9.39	-	-	4.04	-	6.28	-	5.99	-	-	3.99
14	Citronellal	1154	1153	3.71	-	-	-	-	-	-	-	-	-	-	-	-	-	-	-	-	-
15	Isoborneol	1164	1158	-	-	-	-	3.01	-	-	5.02	-	-	2.79	-	-	1.46	2.82	-	-	-
16	Isoneral	1167	1165	-	-	-	-	-	2.68	-	-	2.45	-	-	-	-	-	-	-	2.45	-
17	Borneol	1184	1166	-	-	-	11.35	-	-	-	-	-	-	-	-	2.41	-	4.93	-	-	10.13
18	Terpinen-4-ol	1194	1177	-	-	-	-	-	-	-	-	-	-	-	1.52	-	-	-	0.72	-	-
19	α-Terpineol	1196	1189	-	-	2.66	-	-	2.57	0.59	-	-	1.9	3.9	3.81	3.59	2.29	-	1.49	-	-
20	Isobornyl formate	1231	1235	-	-	-	-	-	-	-	3.23	-	-	-	-	-	-	-	-	-	-
21	Neral	1244	1235	29.14	-	-	-	-	17.23	2.69	9.8	12.34	-	-	-	-	-	-	6.43	10.7	7.68
22	Geranial	1255	1264	53.11	-	-	-	-	33.2	5.11	21.45	24.12	-	-	-	-	-	-	16.97	20.9	15.95
23	Bornyl acetate	1280	1283	-	-	-	2.12	-	-	-	-	-	-	0.88	-	0.68	-	1.9	-		
24	Thymol	1295	1290	-	-	-	-	70.84	-	-	-	45.78	-	-	55.69	-	35.77	33.56	50.46	49.67	37.64
25	α-Terpinyl acetate	1353	1346	-	3.56	2.41	-	-	-	1	-	-	1.65	1.63	-	1.36	1.36	-	-	-	-
26	Ylangene	1387	1368	-	-	-	-	-	-	-	0.33	-	-	-	-	-	-	-	-	-	-
27	Geranyl acetate	1389	1379	4.43	-	-	-	-	6.84	-	-	-	-	-	-	-	-	-	-	-	-
28	Caryophyllene	1417	1420	-	-	-	8.34	-	-	-	4.35	-	-	2.88	-	3.17	-	-	-	-	-
29	Aromandendrene	1446	1440	-	-	-	-	-	1.4	-	-	-	-	0.85	-	-	-	-	-	-	-
30	α-Bisabolene	1530	1540	-	-	-	-	-	-	-	1.75	-	-	-	-	-	-	-	-	-	-
31	Sesquisabinene hydrate	1541	1542	-	-	-	-	-	-	-	0.69	-	-	-	-	-	-	-	-	-	-
32	Spathulenol	1576	1577	-	3.09	0.37	-	-	-	-	-	-	1.77	3.02	-	-	-	-	-	-	-
33	Caryophyllene oxide	1594	1580	-	-	-	-	1.36	-	-	2.42	1.5	-	-	-	-	1.1	-	-	1.5	1.46
34	Globulol	1604	1581	-	-	-	-	-	2.97	-	-	-	-	-	3.2	-	-	-	1.3	-	-
35	Acorenone B	1701	1700	-	-	-	-	-	-	-	-	1.76	-	-	-	-	-	-	-	1.9	1.24
Monoterpene hydrocarbons %				9.59	26.04	24.78	22.92	22.07	25.85	16.9	11.6	10.02	23.39	15.63	33.6	22.8	21.07	20.58	21.99	10.97	10.01
Oxygenated monoterpenes %				90.39	70.79	74.45	68.72	76.56	63.44	83.01	76.85	86.7	74.82	77.59	62.29	73.36	77.81	79.4	76.64	85.56	87.28
Sesquiterpene hydrocarbons %				0	0	0	8.34	0	1.4	0	6.43	0	0	3.73	0	3.17	0	0	0	0	0
Oxygenated sesquiterpenes %				0	3.09	0.37	0	1.36	2.97	0	3.11	3.26	1.77	3.02	3.2	0	1.1	0	1.3	3.4	2.7
Total identified %				99.98	99.92	99.6	99.98	99.99	93.66	99.91	97.99	99.98	99.98	99.97	99.09	99.33	99.98	99.98	99.93	99.93	99.99
Yield %				1.6 ± 0.18 ^bcd^	1.07 ± 0.74 ^abc^	1.78± 0.59 ^cd^	0.85± 0.17 ^ab^	0.53± 0.1 ^a^	0.93 ± 0.05 ^abc^	1.95± 0.45 ^d^	0.71± 0.25 ^a^	1.23 ± 0.24 ^abcd^	1.72± 0.04 ^cd^	1.61 ± 0.06 ^bcd^	0.58± 0.18 ^a^	1.25 ± 0.02 ^abcd^	1.38 ± 0.37 ^abcd^	0.73± 0.1 ^a^	1.14 ± 0.29 ^abcd^	1.62 ± 0.33 ^bcd^	0.95 ± 0.11 ^abc^

CPM-EOs: Combined plant material essential oils; No: Compound number; RI calc.: Calculated retention index relative to the n-alkane series on the HP-5MS capillary column; RI lit.: Literature retention index reported for HP-5MS. Different letters indicate significant differences among groups as determined by one-way ANOVA with Tukey’s HSD test (*p* < 0.05).

**Table 2:** Probit regression results of individual and CPM-EOs against *A. fabae* (24 h post-treatment).

EO Species/Combinations	LC_50_ (µL mL^−1^)	Intercept ± SE	Slope ± SE	χ^2^	*p*-Value
*C. citratus*	3.24	2.044 ± 0.463	−0.631 ± 0.126	25.24	0.000001
*E. camaldulensis*	2.45	0.333 ± 0.229	−0.136 ± 0.035	14.90	0.000114
*E. lehmannii*	2.90	0.580 ± 0.240	−0.200 ± 0.041	24.32	0.000001
*S. rosmarinus*	4.41	1.122 ± 0.257	−0.133 ± 0.034	15.24	0.000095
*T. vulgaris*	3.71	0.837 ± 0.250	−0.226 ± 0.042	29.19	<0.000001
*C. citratus* + *E. camaldulensis*	1.75	1.608 ± 0.468	−0.917 ± 0.220	17.32	0.000032
*C. citratus* + *E. lehmannii*	2.35	2.307 ± 0.627	−0.981 ± 0.240	16.64	0.000045
*C. citratus* + *S. rosmarinus*	2.38	1.494 ± 0.389	−0.628 ± 0.131	23.01	0.000002
*C. citratus* + *T. vulgaris*	1.75	1.608 ± 0.468	−0.917 ± 0.22	17.32	0.000032
*E. camaldulensis* + *E. lehmannii*	3.28	0.905 ± 0.263	−0.276 ± 0.050	30.88	<0.000001
*E. camaldulensis* + *S. rosmarinus*	2.87	1.243 ± 0.320	−0.433 ± 0.082	28.09	<0.000001
*E. camaldulensis* + *T. vulgaris*	1.39	1.352 ± 0.435	−0.972 ± 0.242	16.12	0.000060
*E. lehmannii* + *S. rosmarinus*	2.82	0.777 ± 0.260	−0.276 ± 0.052	28.61	<0.000001
*E. lehmannii* + *T. vulgaris*	1.51	1.430 ± 0.443	−0.945 ± 0.232	16.54	0.000048
*S. rosmarinus* + *T. vulgaris*	1.63	1.515 ± 0.454	−0.927 ± 0.225	16.97	0.000038
*C. citratus* + *T. vulgaris* + *E. camaldulensis*	-	-	-	-	-
*C. citratus* + *T. vulgaris* + *E. lehmannii*	-	-	-	-	-
*C. citratus* + *T. vulgaris* + *S. rosmarinus*	-	-	-	-	-

-: Ternary combinations were not subjected to Probit regression analysis, as they caused 100% mortality at the lowest concentration tested (2 µL mL^−1^), making it impossible to fit a dose–response model. EO = Essential oil; LC_50_ = Lethal concentration; SE = Standard error χ^2^ = Chi-square value.

**Table 3:** Lethal concentration value (LC_50_), co-toxicity coefficient (CTC) and synergistic factors (SF) of CPM-EOs against *A. fabae* (24 h post-treatment).

EO Species/Combination	LC_50_ (µL mL^−1^)	Expected LC_50_ (µL mL^−1^)	CTC	SF vs. A	SF vs. B	Effect Interpretation
*C. citratus* + *E. camaldulensis*	1.75	2.85	162.57	1.85	1.40	Strong synergism
*C. citratus* + *E. lehmannii*	2.35	3.07	130.64	1.38	1.23	Strong synergism
*C. citratus* + *S. rosmarinus*	2.38	3.83	160.71	1.36	1.85	Strong synergism
*C. citratus* + *T. vulgaris*	1.75	3.48	198.57	1.85	2.12	Strong synergism
*E. camaldulensis* + *E. lehmannii*	3.28	2.68	81.58	0.75	0.88	Additive/Slight antagonism
*E. camaldulensis* + *S. rosmarinus*	2.87	3.43	119.52	0.85	1.54	Additive to moderate synergism
*E. camaldulensis* + *T. vulgaris*	1.39	3.08	221.58	1.76	2.67	Strong synergism
*E. lehmannii* + *S. rosmarinus*	2.82	3.66	129.47	1.03	1.56	Strong synergism
*E. lehmannii* + *T. vulgaris*	1.51	3.31	218.81	1.92	2.46	Strong synergism
*S. rosmarinus* + *T. vulgaris*	1.63	4.06	249.08	2.71	2.27	Strong synergism
*C. citratus* + *T. vulgaris* + *E. camaldulensis*	-	-	-	-	-	Highly effective *
*C. citratus* + *T. vulgaris* + *E. lehmannii*	-	-	-	-	-	Highly effective *
*C. citratus* + *T. vulgaris* + *S. rosmarinus*	-	-	-	-	-	Highly effective *

Interaction types were interpreted using both the co-toxicity coefficient (CTC) and the synergistic factor (SF). A CTC value > 120 indicates synergism, 80–120 indicates an additive effect, and <80 indicates antagonism. Similarly, an SF > 1 suggests synergism, SF = 1 indicates additivity, and SF < 1 suggests antagonism. *: The inability to estimate LC_50_ values due to 100% mortality at minimal doses (2 µL mL^−1^), indicating strong synergistic effects. EO = Essential oil; LC_50_ = Lethal concentration.

**Table 4:** Pearson correlation coefficients (r) and *p*-values between EOs compounds and LC_50_ values.

Chemical Compound	r	*p*-Value	Significance
α-Pinene	0.3942	0.105	NS
Camphene	0.3829	0.117	NS
β-Pinene	0.4248	0.079	NS
β-Myrcene	0.0837	0.741	NS
3-Carene	0.0514	0.839	NS
α-Terpinene	−0.0325	0.898	NS
*p*-Cymene	−0.3312	0.179	NS
Eucalyptol	0.4664	0.051	NS
γ-Terpinene	−0.4387	0.069	NS
Sabinene hydrate	0.2340	0.350	NS
Linalool	−0.2652	0.287	NS
Pinocarveol	−0.1045	0.680	NS
Camphor	0.2265	0.366	NS
Citronellal	0.2272	0.364	NS
Isoborneol	0.1521	0.547	NS
Isoneral	−0.3578	0.145	NS
Borneol	0.0884	0.727	NS
Terpinen-4-ol	−0.3383	0.170	NS
α-Terpineol	0.0019	0.994	NS
Isobornyl formate	0.0359	0.888	NS
Neral	−0.1616	0.522	NS
Geranial	−0.2101	0.403	NS
Bornyl acetate	0.3534	0.150	NS
Thymol	−0.5018	0.034	*
α-Terpinyl acetate	0.2596	0.298	NS
Ylangene	0.0359	0.888	NS
Geranyl acetate	0.0370	0.884	NS
Caryophyllene	0.5267	0.025	*
Aromandendrene	−0.0144	0.955	NS
α-Bisabolene	0.0359	0.888	NS
Sesquisabinene hydrate	0.0359	0.888	NS
Spathulenol	0.2442	0.329	NS
Caryophyllene oxide	−0.2312	0.356	NS
Globulol	−0.3233	0.191	NS
Acorenone B	−0.5119	0.030	*

* Indicates statistically significant correlation (*p* < 0.05); NS = not significant.

**Table 5:** Compounds with VIP scores greater than 1 in the PLS regression analysis.

Rank	Variable Number	Chemical Compound	VIP Score
1	35	Acorenone B	1.762
2	28	Caryophyllene	1.663
3	24	Thymol	1.458
4	14	Citronellal	1.457
5	8	Eucalyptol	1.398
6	11	Linalool	1.379
7	9	γ-Terpinene	1.286
8	3	β-Pinene	1.246
9	2	Camphene	1.161
10	1	α-Pinene	1.149
11	6	α-Terpinene	1.112
12	4	β-Myrcene	1.098
13	7	*p*-Cymene	1.096
14	13	Camphor	1.045
15	16	Isoneral	1.039
16	23	Bornyl acetate	1.029
17	18	Terpinen-4-ol	1.028

The top 17 compounds ranked by their variable importance in projection (VIP). Variable numbers correspond to those used in the chemical composition table (Table 1).

**Table 6:** Plant species, used part, period and harvesting sites.

Species	Used Part	Harvesting Period	Location
*C. citratus*	Leaves	March–April/2025	Kairouan (35.129849° N, 10.142680° E)
*E. camaldulensis*	Leaves		Sejnane (37.157730° N, 9.135421° E)
*E. lehmannii*	Leaves		Ain drahem (36.876936° N, 8.695837° E)
*S. rosmarinus*	Aerial parts		Tborsok (36.499551° N, 9.318492° E)
*T. vulgaris*	Aerial parts		Tborsok (36.497912° N, 9.319307° E)

## Data Availability

The original contributions presented in this study are included in the article/Supplementary Materials. Further inquiries can be directed to the corresponding author.

## Supplementary Materials

The following supporting information can be downloaded at: https://www.mdpi.com/article/10.3390/molecules31060945/s1, Table S1: Factor loadings (PC1, PC2 and PC3) for individual and CPM-EOs based on PCA; Table S2: Summary of PLS regression relating chemical composition to LC_50_; Table S3: VIP scores of all identified chemical compounds from PLS regression analysis; Figure S1: Schematic diagram of the experimental design. Essential oils were extracted from individual plant species and from binary (1:1) and ternary (1:1:1) mixtures of plant material prior to hydro-distillation. Extracted oils were analyzed chemically and tested for insecticidal activity, followed by statistical and synergy analyses. Species included *C. citratus*, *E. camaldulensis*, *E. lehmannii*, *S. rosmarinus*, and *T. vulgaris*. EO = essential oil; GC-MS = gas chromatography–mass spectrometry; HCA = hierarchical cluster analysis; PCA = principal component analysis; PLS = partial least squares.
