# Supplementary material for: Synergistic and Additive Interactions in Essential Oils Obtained from Combined Plant Materials: Enhanced Control of Insect Pests
Source: Molecules. 2026 Mar 12;31(6):945. doi: 10.3390/molecules31060945 (PMC13029542; doi:10.3390/molecules31060945)
Supplement: Supplementary file 1 [file molecules-31-00945-s001.zip › molecules-4024446-supplementary.pdf]

Table S1. Factor loadings (PC1, PC2 and PC3) for individual and CPM-EOs based on PCA

Essential oils	PC1	PC2	PC3
Individual Oils			
C. citratus	–0.03	–0.26	**0.94**
E. camaldulensis	**–0.91**	0.36	0.00
E. lehmannii	**–0.89**	0.37	–0.01
S. rosmarinus	**–0.88**	0.38	–0.01
T. vulgaris	–0.29	**–0.89**	–0.32
Binary Mixtures			
C. citratus + *E. camaldulensis*	0.00	–0.29	**0.88**
C. citratus + *E. lehmannii*	**–0.93**	0.33	0.07
C. citratus + *S. rosmarinus*	**–0.70**	0.12	**0.63**
C. citratus + *T. vulgaris*	–0.28	**–0.92**	0.18
E. camaldulensis + *E. lehmannii*	**–0.92**	0.36	0.00
E. camaldulensis + *S. rosmarinus*	**–0.91**	0.37	0.00
E. camaldulensis + *T. vulgaris*	–0.27	**–0.87**	–0.30
E. lehmannii + *S. rosmarinus*	**–0.90**	0.38	–0.01
E. lehmannii + *T. vulgaris*	**–0.88**	–0.36	–0.25
S. rosmarinus + *T. vulgaris*	**–0.84**	–0.42	–0.27
Ternary Mixtures			
C. citratus + *T. vulgaris* + *E. camaldulensis*	–0.28	**–0.94**	0.01
C. citratus + *T. vulgaris* + *E. lehmannii*	–0.29	**–0.94**	0.09
C. citratus + *T. vulgaris* + *S. rosmarinus*	–0.52	**–0.80**	0.07

Table S2. Summary of PLS regression relating chemical composition to LC_{50}

Component	R^2X	R^2X (Cumulative)	Eigenvalue	R^2Y	R^2Y (Cumulative)	Q^2	Limit	Q^2 (Cumulative)	Significance	Iterations
1	0.2186	0.2186	6.73	0.4162	0.4162	0.1417	0.00	0.1417	S	1
2	0.1061	0.3247	1.91	0.1994	0.6157	-1.1516	0.00	-0.8467	NS	1

Table S3. VIP scores of all identified chemical compounds from PLS regression analysis.

Rank	Variable number	Chemical compound	VIP score
1	35	Acorenone B	1.762
2	28	Caryophyllene	1.663
3	24	Thymol	1.458
4	14	Citronellal	1.457
5	8	Eucalyptol	1.398
6	11	Linalool	1.379
7	9	γ -Terpinene	1.286
8	3	ß-Pinene	1.246
9	2	Camphene	1.161
10	1	α-Pinene	1.149
11	6	α-Terpinene	1.112
12	4	ß-Myrcene	1.098

13	7	*p*-Cymene	1.096
14	13	Camphor	1.045
15	16	Isoneral	1.039
16	23	Bornyl acetate	1.029
17	18	Terpinen-4-ol	1.028
18	34	Globulol	0.939
19	27	Geranyl acetate	0.927
20	12	Pinocarveol	0.909
21	25	α-Terpinyl acetate	0.865
22	32	Spathulenol	0.729
23	10	Sabinene hydrate	0.687
24	33	Caryophyllene oxide	0.671
25	22	Geranial	0.670
26	21	Neral	0.657
27	17	Borneol	0.654
28	15	Isoborneol	0.479
29	5	3-Carene	0.473
33	20	Isobornyl formate	0.363
33	26	Ylangene	0.363
33	30	α-Bisabolene	0.363
33	31	Sesquisabinene hydrate	0.363
34	19	α-Terpineol	0.357
35	29	Aromandendrene	0.075

Variable numbers correspond to those used in the chemical composition table (Table 1). Compounds with identical VIP scores share the same rank.

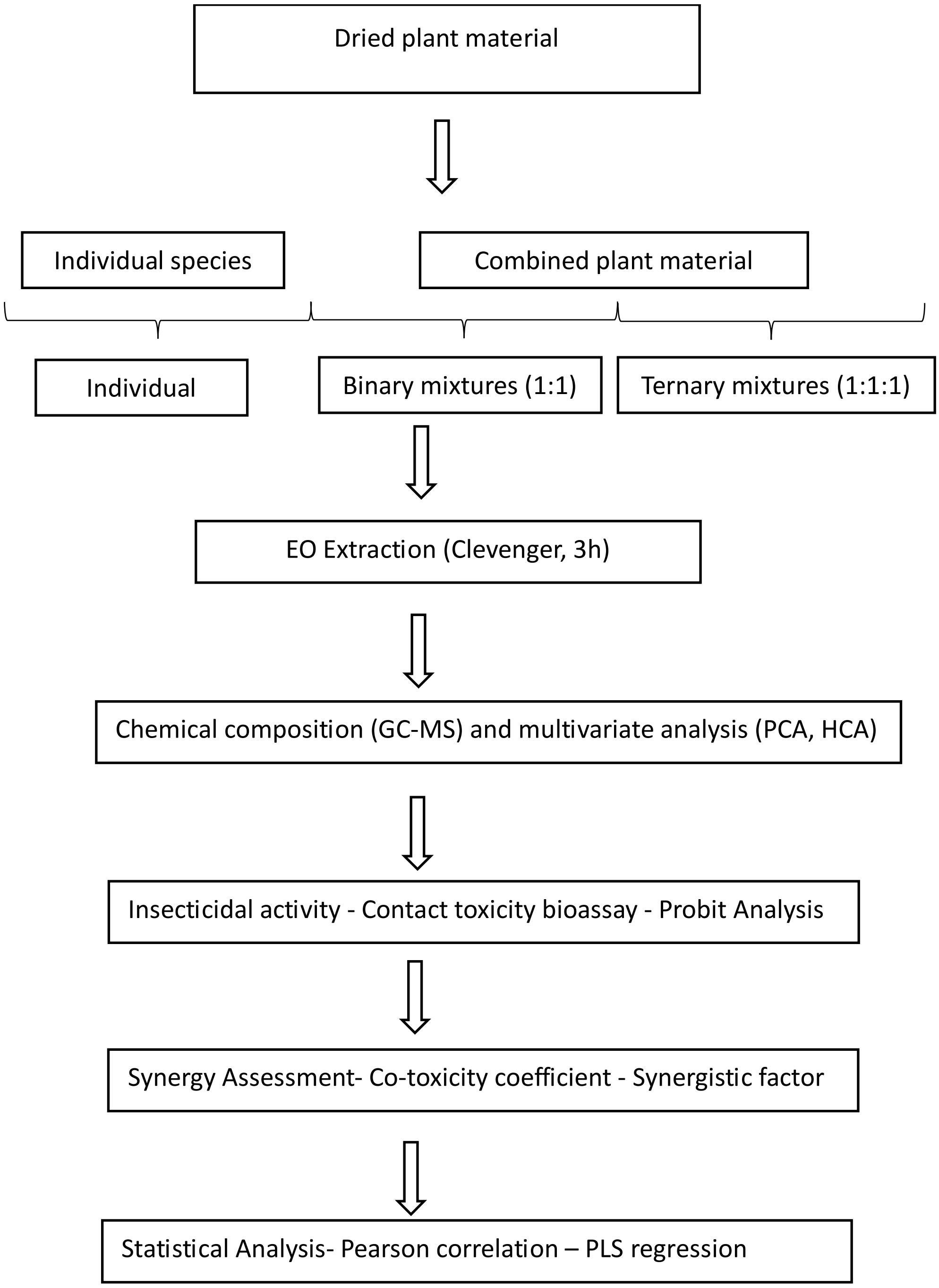

Figure S1. Schematic diagram of the experimental design. Essential oils were extracted from individual plant species and from binary (1:1) and ternary (1:1:1) mixtures of plant material prior to hydro-distillation. Extracted oils were analyzed chemically and tested for insecticidal activity, followed by statistical and synergy analyses. Species included *C. citratus*, *E. camaldulensis*, *E. lehmannii*, *S. rosmarinus*, and *T. vulgaris*. EO = essential oil; GC-MS = gas chromatography–mass spectrometry; HCA = hierarchical cluster analysis; PCA = principal component analysis; PLS = partial least squares.
